# The potential impact of RNA splicing abnormalities on immune regulation in endometrial cancer

**DOI:** 10.1038/s41419-025-07458-7

**Published:** 2025-03-03

**Authors:** Minyue Cao, Jiayu Yan, Yan Ding, Yiqin Zhang, Yihan Sun, Genyi Jiang, Yanli Zhang, Bilan Li

**Affiliations:** Shanghai Key Laboratory of Maternal Fetal Medicine, Shanghai Institute of Maternal-Fetal Medicine and Gynecologic Oncology, Shanghai First Maternity and Infant Hospital, School of Medicine, Tongji University, Shanghai, 200092 China

**Keywords:** Endometrial cancer, Immunosurveillance, RNA splicing

## Abstract

RNA splicing controls the post-transcriptional level of gene expression, allowing for the synthesis of many transcripts with various configurations and roles. Variations in RNA splicing regulatory factors, including splicing factors, signaling pathways, epigenetic modifications, and environmental factors, are typically the origin of tumor-associated splicing anomalies. Furthermore, thorough literature assessments on the intricate connection between tumor-related splicing dysregulation and tumor immunity are currently lacking. Therefore, we also thoroughly discuss putative targets associated with RNA splicing in endometrial cancer (EC) and the possible impacts of aberrant RNA splicing on the immune control of tumor cells and tumor microenvironment (TME), which contributes to enhancing the utilization of immunotherapy in the management of EC and offers an alternative viewpoint for the exploration of cancer therapies and plausible prognostic indicators.

## Facts

- Essential role of RNA splicing: RNA splicing is essential for gene expression regulation, impacting various transcript configurations and functions.
- Origin of tumor-associated anomalies: variations in splicing factors, signaling pathways, epigenetic modifications, and environmental factors cause tumor-associated splicing anomalies.
- Research gaps in splicing and immunity: current research lacks a comprehensive understanding of the link between tumor-related splicing dysregulation and tumor immunity.
- RNA splicing in EC: RNA splicing plays a significant role in EC, affecting tumor cell immunity and the TME.
- Potential for immunotherapy: aberrant RNA splicing in many tumors can influence immunotherapy efficacy and provide new perspectives for cancer treatment and prognosis.

## Open questions

- Mechanisms of splicing regulation: How do splicing factors, signaling pathways, epigenetic modifications, or environmental factors specifically regulate RNA splicing in the context of cancer?
- Impact on tumor immunity: What is the precise impact of aberrant RNA splicing on the immune response within the TME?
- Therapeutic targets: Which RNA splicing targets in EC are most promising for developing new cancer therapies?
- Prognostic indicators: How can RNA splicing patterns be used as prognostic indicators in EC?
- Immunotherapy enhancement: How can understanding RNA splicing improve the design of immunotherapies for EC?

## Introduction

The third most common tumor in the female reproductive system, endometrial cancer (EC) accounts for 2.1% of all cancer incidences, according to the global cancer statistics of 2022 [1]. Traditionally, EC is classified into type I (estrogen-dependent) and type II (non-estrogen-dependent) based on its pathogenesis and biological behavior characteristics [2], which ignores the molecular biological features of the tumor, essential for better understanding the tumor’s biological behavior and directing customized treatment. Therefore, EC is classified into four subtypes by the Cancer Genome Atlas (TCGA): POLE ultramutated, microsatellite instability high (MSI-H), copy-number low (CNL), and copy-number high (CNH), based on genomic, transcriptomic, proteomic, and other data. Targeted combination immunotherapy has shown good efficacy in advanced or recurrent MSI-H/dMMR type EC [3], because this type has a higher tumor mutation burden and immune cell infiltration, exhibiting a “hot” tumor microenvironment (TME), making them ideal candidates for immunotherapy [4]. Determining potential targets for immunotherapy is therefore imperative.

In eukaryotic organisms, RNA splicing, which entails removing non-coding segments (introns) from pre-mRNA and connecting coding sequences (exons) to generate mature mRNA, is an essential procedure in gene expression during the post-transcriptional modification phase. Significantly, splicing mistakes can produce abnormal protein products that can either cause or worsen certain disorders and malignancies [5–7]. Compared with healthy tissues surrounding cancer, most tumor tissues show substantial splicing variations that can be generated by various mechanisms and are closely associated with the onset and progression of cancer [8].

Though research on this area is still in its early stages, we discuss the regulatory mechanisms of RNA splicing, putative targets related to RNA splicing in EC, and the potential consequences of RNA mis-splicing on tumor immune regulation in this review. This provides an important direction for future RNA-splicing-based EC studies.

## Processes and types of RNA splicing

**Fig. 1:**
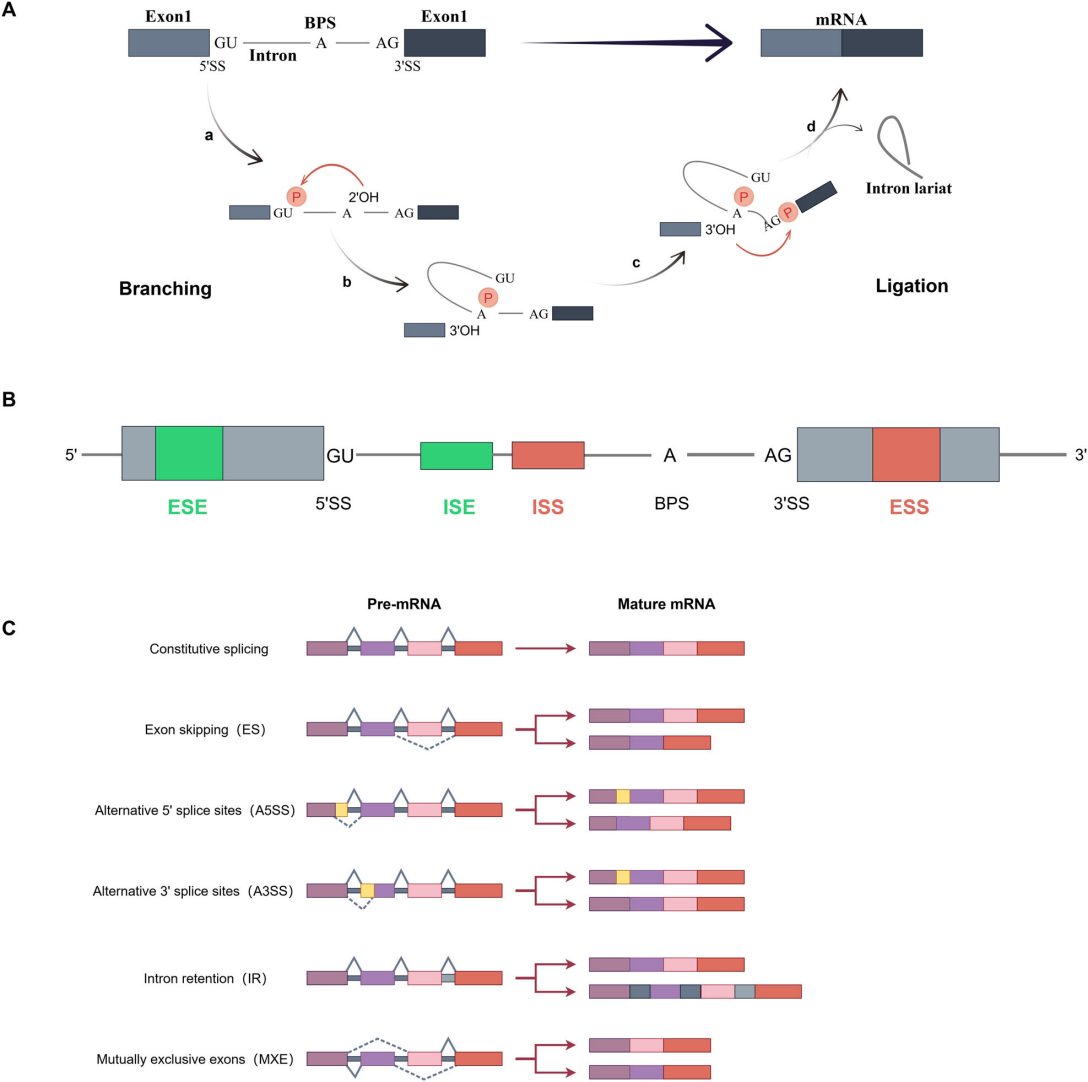
Processes and types of RNA splicing. **A** RNA splicing process involves two primary steps: branching and ligation. BPS branch point site. **B** Spliceosomes recognize exon splicing enhancer (ESE), exon splicing silencer (ESS), intron splicing enhancer (ISE), and intron splicing silencer (ISS) splice sites in pre-RNA. **C** RNA splicing is divided into AS and CS based on the diversity of splicing products and AS is divided into five types: ES, A5SS, A3SS, IR, and MXE. By Figdraw.

Based on precise cutting and connection at specific sites, ligation and branching are two fundamental RNA splicing processes (Fig. 1A). The 5’ end of the intron is connected to the branch adenosine in the first step, which involves cutting the 5’ splicing site (5’ss). The 3’-OH group of the 5’ exon cuts the 3’ splicing site (3’ss) in the second step and connects it to the 5’ and 3’ exons [9]. These events rely on a complex called a spliceosome, which consists of several splicing factor proteins and small nuclear RNAs (U1, U2, U4, U5, and U6) and can identify the appropriate splicing sites and perform cutting and connecting [10], such as splicing enhancers, splicing silencers, exons, and introns (Fig. 1B).

RNA splicing is commonly categorized into two primary types: constitutive splicing (CS) and alternative splicing (AS), based on the diversity of products (Fig. 1C). Complying with the GU-AG splicing site rule, AS allows the creation of distinct mature mRNA splice variants from a single pre-mRNA, each of which can be spliced differently inside the cell. The five primary splicing modes in this process are exon skipping (ES), alternative 5’ splice site (A5SS), alternative 3’ splice site (A3SS), intron retention (IR), and mutually exclusive exons (MXE) [11]. Up to 90% of human genes go through AS in normal physiological conditions, resulting in various protein products and amplifying the functional diversity of the genome [12]. In contrast to AS, CS involves the splicing of pre-mRNA into stable, consistent mRNA, following the GT-AG splicing site rule, where all pre-mRNA for a certain gene is uniformly removed of introns and joined to exons. This is important for maintaining the constancy of proteins in biological processes and cell functions [13].

## Regulation of RNA splicing

**Fig. 2:**
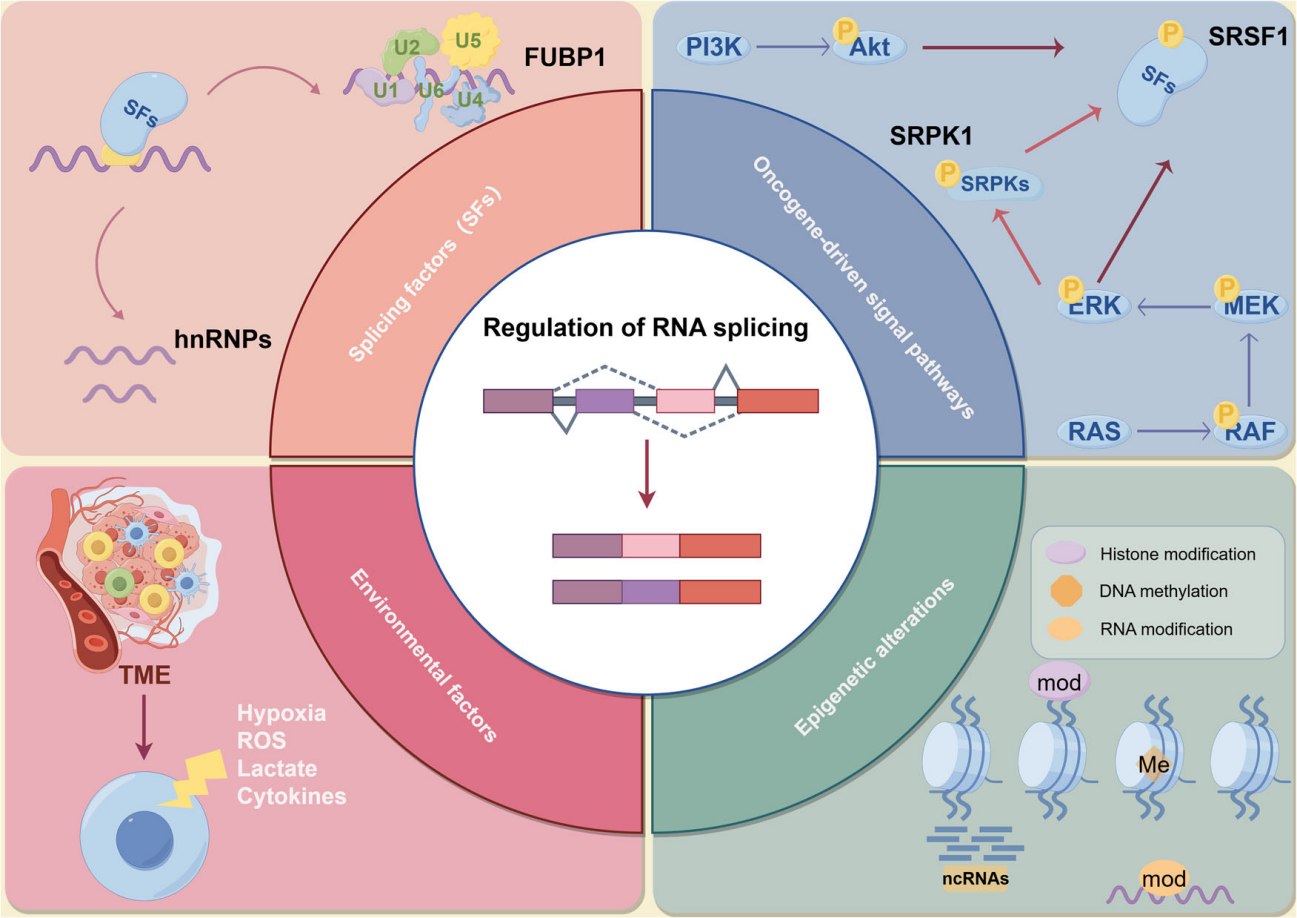
Clockwise, starting from top left: SFs; top right: oncogene-driven signal pathways; bottom right: epigenetic alterations; bottom left: environmental factors. By Figdraw.

The precise results of RNA splicing depend on how the spliceosome is assembled and how the splicing process is executed. Thus, this review summarizes RNA splicing regulatory factors, including splicing factors, oncogenic signaling pathways, epigenetic alternations, and environmental factors (Fig. 2).

### Splicing factors (SFs)

SFs identify certain splicing sites during RNA splicing and facilitate spliceosome construction and splicing reactions. One important SF that bridges lengthy intron splicing locations is the multi-domain RNA-binding protein FUBP1. By attaching itself to the as-yet-undefined cis-acting elements, it stabilizes the important constituents U2AF2 and SF126 at the 3’ss by utilizing its polyvalent binding sites in the disordered area [14]. Besides, SFs play a regulatory role in controlling AS. Taking the heterogeneous nuclear ribonucleoprotein (hnRNP) family as an example, hnRNP H and F trigger HRAS exon 5 splicing by binding to splicing enhancer sites in introns, hence contributing to prostate cancer cell proliferation [15]. HnRNP H promotes human papillomavirus type 16 (HPV16) E6^7 transcript expression by binding to numerous GGG motifs downstream of splicing site SD880 [16].

Due to the critical role in controlling splicing, the deregulation of SFs in cancer results in splicing mistakes, accelerating the tumor process or encouraging the conversion of normal cells into malignant ones. Mutations in SF3B1 are closely associated with myelodysplastic syndromes and other hematologic malignancies [17]. Additionally, excessive SF3B1 expression in EC cells may disrupt KSR2’s ability to promote the proliferation of EC cells by causing erroneous splicing of several genes and altering the maturation process of KSR2 mRNA [18].

### Oncogene-driven signal pathways

A number of crucial traits that normal cells display as they progressively change into tumor cells provide a strong basis for the emergence and progression of cancer [19], which largely depends on oncogene-driven signal pathways, offering prospective targets for the creation of tailored medications in the therapeutic domain.

The RAS/RAF/ERK signaling pathway involves RAS proteins activated by receptor tyrosine kinases (RTKs), which in turn sequentially activate three mitogen-activated protein kinases (RAF, MEK, and ERK). ERK enters the cell nucleus, activates downstream targets, and achieves fine-tuned regulation of cellular behavior. The phosphorylation of SFs can result from activation of this signaling pathway, which can either encourage or hinder the inclusion of particular exons [20]. Sam68, a novel target of the ERK pathway, can be phosphorylated and activated in T cells, increasing the retention of the exonv5 in CD44 mRNA and thereby raising the invasiveness of certain tumors [21]. Furthermore, as a nuclear RNA-binding protein, Sam68 can be phosphorylated and activated by ERK in T cells, increasing its RNA-binding activity [21, 22]. Colon cancer has also been found to activate the similar mechanism for Sam68, which binds phosphorylated Sam68 to the 3’ untranslated region (3’UTR) of the SRSF1 transcript and facilitates intron inclusion, which is necessary for the synthesis of full-length SRSF1 transcripts [23]. However, when Sam68 is specifically phosphorylated by CDK1 at the T33 and T317 sites, its RNA-binding activity is weakened, its cellular localization is controlled, and its AS activity is reduced [24]. This implies that the RNA-binding activity of Sam68 is affected differently by phosphorylation at various locations. According to recent research, Sam68 is increased in EC cell lines and clinical samples and is crucial for encouraging cell proliferation [25]. Therefore, whether Sam68 promotes cell proliferation in EC by affecting certain SFs requires further research.

The first step in the PI3K/AKT signaling pathway is PI3K activation, which produces PIP3, the second messenger, to attract and activate Akt. Activated Akt can directly phosphorylate serine/arginine-rich splicing factors (SRSFs) [26]. To produce the anti-apoptotic casp-9b isoform, Akt can directly phosphorylate SRSF1, mediating the exclusion of casp9’s exon3-6 [27]. Furthermore, it can cause SR protein-specific kinases (SRPKs) to autophosphorylate [28], which sends a signal to the nucleus indirectly and controls the phosphorylation state of SRSFs and AS. In order to facilitate SRPK1’s transition from a Hsp70-containing complex to a Hsp90-containing complex, activated Akt causes autophosphorylation of SRPK1, which in turn promotes SRPK1’s nuclear translocation and the hyperphosphorylation of SRSFs [28]. According to tumor cell migration screening, SRPK1 is essential for breast cancer metastasis [29]. Overexpression of SRPK1, which can be regulated by tumor suppressor WT1 transcriptionally, induces the pro-angiogenic VEGFA-165a transcript by hyperphosphorylating SRSF1 [30]. Furthermore, targeting SRPK1 and EGFR or IGF1R at the same time can synergistically inhibit EC cell growth and survival, indicating that combination therapy may be a potential therapeutic option [31].

However, AS variants can also regulate the transmission of signaling pathways in turn. By triggering the MAPK/ERK/MMP2 signaling cascade, the short splicing variation of DCLK1 (DCLK1-S) might encourage the growth and invasion of esophageal squamous cell carcinoma cells, and the ERK1/2 inhibitor SCH772984 can lessen these functions [32]. Additionally, a novel splicing variant of the estrogen receptor-α (ER-α30) functions as an oncogenic factor in triple-negative breast cancer (TNBC), acting as an appealing candidate for TNBC therapy by triggering the PI3K/AKT signaling pathway to promote drug resistance, cell proliferation, invasion, and metastasis [33].

### Epigenetic alterations

RNA splicing and epigenetic modifications are frequently dysregulated in various cancers, according to plenty of studies [34, 35]. Nevertheless, there is presently a paucity of research that links these two phenomena (Table 1).

**Table 1:** Epigenetic alterations and RNA splicing.

Epigenetic alterations	Impact on RNA splicing	Examples
DNA methylation	Directly affects the recognition of exons	EGFR, HPCAL1-SV and RNASET2-SV [39] The exon1 of the SFRP1 gene [40] The exon12 of the MBD1 gene [41]
	Indirectly affects the inclusion and exclusion of exons	The exon7 of the GCK gene [42]
Histone modification	Affects the identification of splice sites and the splicing efficiency	H3K36me3 and H3K27me3 [43]
	Affects the AS process	H3K36me—the intron2 retention in DVL2 mRNA [44] H3K36me3—the ES of IL-18 [45]
ncRNA	MiRNA targeted inhibition of SFs	MiR-30a-5p and miR-181a-5p—SRSF7 [26] MiR-802—SRSF9 [46] MiR-28 and miR-505—SRSF1 [47] MiR-10a and miR-10b—SRSF1 [48] MiR-10b—U6 snRNA [49]
	LncRNA influences the stability, subcellular localization, or interactions between SFs and pre-mRNAs	LncRNA CRNDE—SRSF6 [50] LncRNA H19—SRSF1 [51] LncRNA MALAT1 and LINC01133—SRSFs’ nuclear localization [52, 53] LncRNA CACClnc—YB1 and U2AF65 [54] LncRNA PLANE, MIR99AHG and LINC01089—hnRNPM and PTBP1 [55–57]
	LncRNA encodes splicing regulatory protein	LncRNA LOC90024 —the “cancerous” Sp4 splice variant [58]
RNA modification	M6A readers participate in the regulation of AS	YTHDC1—lncHOXB-AS3 splice variant NR_033205.1 and RBM4-S variant [62, 63] HnRNPC—TAF8S subtype [65]
	Non-m6A modifications	2’-O-methylation modification—U12-type intron splicing [66]
SNP	Disrupts splice sites	PKD1 (c.7866C > A) [67] TCHP (rs74416240) [68] FARP1 (rs677031-G) [69] PSMD13 (rs7128029 A < G) [70] ELP2 (rs1785932-T) [71]
	Creates new splice sites	PTEN (rs786204926-G) [72] GTPBP3 (c.809-1_809delinsA) [73]
	Affects the splice adjustment elements	PKD1 (c.7866C > A,c.7960A > G, c.7979A > T, c.7987C > T, c.11346C > T and c.11393C > G) [67] ACADM (c.351A > C, c.325A > C, c.324T > C and c.326G > C) [74] IL7R (rs6897932) [75]
	Alters RNA secondary structure	EGR1 (rs1995158) [76]

#### DNA methylation

DNA methylation mostly happens at methylation sites called CpG islands, which are situated between cytosine (C) and guanine (G). Numerous CpG sites are strongly connected to exon expression in different types of cancer based on TCGA data. These CpG locations exhibit a stronger association with patient prognosis than CpG sites unrelated to exon expression [36]. On the one hand, CpG methylation may have a direct impact on exon recognition since exons have a higher frequency of methylation than introns [37, 38]. Aberrant splice variants with specific high expression were identified in the TCGA and Clinical Proteomic Tumor Analysis Consortium (CPTAC) cohorts of clear cell renal cell carcinoma. Among these, there was a negative correlation between gene-specific CpG methylation and the expression of EGFR, HPCAL1-SV, and RNASET2-SV [39]. Additionally, the loss of SFRP1 mRNA and protein expression in pancreatic cancer is significantly influenced by a core CpG island (CGI2) that covers the transcription start site and the first half of exon1 of the SFRP1 gene, which suggests that CGI2 may influence exon1 recognition through methylation of exon1 [40]. The splicing of exon12 of MBD1 is linked to the CpG island methylator phenotype generated by IDH mutation in gliomas, which is characterized by promoter hypermethylation and gene silence [41]. CpG methylation, on the other hand, may have an indirect effect on exon7 inclusion or exclusion of the GCK gene by altering the creation and stability of G-quadruplexes and interacting with transcription factors such as EGR1. These alterations may have a substantial impact on the development of metabolic disorders and hepatocellular cancer [42]. This implies that DNA methylation might control whether exons are included or excluded.

#### Histone modification

Histone modification can impact the efficiency of splicing and the spliceosome’s ability to recognize splicing sites. Distinct enrichment regions surrounding splicing sites can be formed by the histone modification patterns of H3K36me3 and H3K27me3, which may aid the spliceosome in identifying splicing sites and enhance splicing efficiency [43]. Additionally, it can have an effect on AS. Under normal conditions, because the intron2 of DVL2 mRNA contains a premature termination codon (PTC), the transcript is cleared through the nonsense-mediated mRNA decay pathway. Nonetheless, a reduction in H3K36 methylation levels is seen in colon cancer, which may lessen the retention of intron2 in DVL2 mRNA and increase the expression of DVL2 transcripts, enhance Wnt signaling transduction, and encourage malignant transformation [44]. Furthermore, H3K36me3 can induce ES of IL-18 by recruiting PTBP3, resulting in the elevation of ΔIL-18 levels exclusively produced in malignancies, which is directly associated with immune escape [45].

#### Non-coding RNA (ncRNA)

Through partial complementary pairing, microRNAs (miRNAs) bind to the 3’UTR of target mRNAs, preventing or accelerating mRNA translation and controlling gene expression. MiRNAs can target SFs as their downstream regulatory sites. MiR-30a-5p and miR-181a-5p have the ability to reduce the expression of SRSF7 in renal cell carcinoma, which alters oncogenes, tumor suppressors, and apoptotic regulating factors’ splicing patterns [26]. MiR-802 inhibits cell proliferation and induces apoptosis in cervical cancer by targeting SRSF9 [46]. Not inhibited by leukemia/lymphoma-associated factor, miR-28 and miR-505 can target and inhibit the SRSF1, causing cellular senescence or apoptosis in mouse embryonic fibroblasts [47]. The elevated production of miR-10a and miR-10b in retinoic acid-induced neuroblastoma cells can inhibit SRSF1, which in turn promotes the final differentiation of neuroblastoma cells [48]. Moreover, splicing processes and miRNAs have been found to unexpectedly interact in glioblastoma: miR-10b can control the amount, stability, modification, and conformation of U6 snRNA, which in turn impacts the splicing pattern of CDC42, accelerating tumor progression [49].

Long non-coding RNAs (lncRNAs) control the splicing process by influencing the stability, subcellular localization, or interactions between SFs and pre-mRNAs. LncRNA CRNDE directly binds to SRSF6, decreasing the protein’s stability and controlling the AS of PICALM mRNA [50]. LncRNA H19 can also competitively bind to miR-107, releasing YTHDC1 mRNA and interacting with YTHDC1 protein to modulate SRSF1 stability, eventually influencing IL-6 and IL-10 AS and driving tumor growth [51]. Certain lncRNAs, such as MALAT1 and LINC01133, have the ability to influence AS by controlling SRSFs’ nuclear localization [52, 53]. CACClnc may bind to U2AF65 and YB1 selectively, facilitating their interaction and regulating the AS of RAD51 mRNA as a result [54]. To control the AS of NCOR2, SMARCAD1, and DIAPH3, hnRNPM and PTBP1 interact with lncRNA PLANE, MIR99AHG, and LINC01089 [55–57]. Interestingly, some lncRNAs actually have the ability to encode microproteins or tiny peptides that could be involved in aberrant RNA splicing. By strengthening SRSF3’s binding to exon3 of the transcription factor Sp4, the splicing regulatory protein produced by lncRNA LOC90024 can cause the production of the “cancerous” Sp4 splice variant [58].

#### RNA modification

The m6A modification, prevalent in eukaryotic cells, has an impact on almost all facets of mRNA processing. The regulatory network for m6A modification is dynamically reversible, with “writers” (methyltransferases), “erasers” (demethylases), and “readers” (recognition proteins) making up the network. Three main families of m6A “readers” are currently identified: hnRNPs, insulin-like growth factor-2 mRNA-binding proteins (IGF2BPs), and YT521-B homology (YTHs) [59]. An increasing amount of evidence indicates that m6A “readers”, who frequently collaborate with SRSFs, are also involved in the regulation of AS [60]. YTHDC1 selectively binds SRSF3 via a m6A-dependent mechanism to facilitate the retention of certain exons in m6A-modified mRNAs [61]. It can upregulate the levels of the lncHOXB-AS3 splice variant NR_033205.1 to cause leukemia stem cells to self-renew, which in turn accelerates the progression of acute myeloid leukemia [62]. The tumor-promoting RBM4-S variant is produced in lung cancer cells via the m6A-YTHDC1 complex, which facilitates exon3 skipping of RBM4 mRNA by attracting AURKA-mediated hnRNPK [63]. Being both a SF and a m6A “reader”, hnRNPC has the potential to bind to m6A-modified mRNA and use the m6A regulatory mechanism to influence the stability of the secondary structure of mRNA, improve protein-RNA interaction, and ultimately control the AS of targeted mRNAs [64]. It promotes the synthesis of the metastatic-promoting TAF8S subtype by regulating the AS of TAF8 through the previously described mechanism in pancreatic ductal adenocarcinoma (PDAC) [65].

Additionally, recent studies have demonstrated how crucial 2’-O-methylation modification is to U12-type intron splicing. NcRNA bktRNA1 can interact with the minor spliceosome U12 snRNA, accurately guiding the methyltransferase to add methylation modifications at specific sites, which is crucial for maintaining the accuracy of U12 splicing [66].

#### Single nucleotide polymorphism (SNP)

Splice sites are typically enriched with specific nucleotide sequences. The introduction of SNPs may diminish or enhance these signals, leading to erroneous assembly of the spliceosome. SNPs can alter the critical nucleotide sequences at the 5’ or 3’ss, rendering them unrecognizable by the spliceosome and resulting in ES or IR. For instance, the c.7866C > A located at the 5’ end of exon21 in the PKD1 gene may alter the recognition capability of the site, leading to partial skipping of exon 21 [67]. The SNP rs74416240, situated at the last nucleotide of exon4 in the TCHP gene, disrupts the 5’ss of TCHP exon4 and results in IR [68]. The rs677031-G variant promotes AS of the FARP1 gene exon20, reducing the expression level of the long transcript FARP1-011, thereby influencing the progression of non-small cell lung cancer [69]. The SNP rs7128029 A < G, which mediates skipping of PSMD13 exon2, is significantly associated with an increased risk of endometrial cancer [70]. The rs1785932-T allele identified in PDAC promotes AS of ELP2 exon 6, leading to a disproportionate ratio of full-length ELP2 isoforms to truncated ELP2 isoforms, thereby reducing the risk of PDAC [71]. Conversely, SNPs can also create a novel splice site where none existed, causing adjacent sequences to be mistakenly identified as exons and introducing additional sequences into mature mRNA. This phenomenon, observed in some diseases, results in the production of aberrant proteins. For example, the G-allele preference for rs786204926 produces a new PTEN mutant, which inserts 18 bp from intron4, is more prone to dephosphorylation, and leads to chemotherapy sensitivity in breast cancer patients via the PI3K-AKT signaling pathway [72]. The newly discovered c.809-1_809delinsA mutation in the GTPBP3 gene affects splicing of exon7, resulting in an 8-base pair deletion on the left side of exon7 and exon7 skipping, leading to the emergence of an abnormal truncated protein [73].

Splicing is not solely reliant on the splice sites themselves but also requires a variety of cis-acting elements, including exon splicing enhancers (ESEs), exon splicing silencers (ESSs), intron splicing enhancers (ISEs), and intron splicing silencers (ISSs). By creating a substantial imbalance in the ratio of ESEs to ESSs, variants in the PKD1 gene (c.7866C > A, c.7960A > G, c.7979A > T, c.7987C > T, c.11346C > T, and c.11393C > G) can interfere with normal pre-mRNA splicing and prevent the inclusion of exon21 to varying degrees, resulting in in-frame deletions [67]. SNPs in two distinct ESSs within exon5 of the ACADM gene (such as c.351A > C, c.325A > C, c.324T > C, and c.326G > C) disrupt ESSs and alter the binding of splicing factors (SFs), thereby affecting the recognition of splice sites [74]. The disease-associated allele of SNP rs6897932 in exon6 of IL7R intensifies ESSs and increases the skipping of exon6, leading to an elevated production of soluble IL7R [75].

Furthermore, SNPs can induce significant alterations in the secondary structure of RNA, potentially impeding the binding of splicing factors or revealing previously masked splice sites. For instance, the rs1995158 SNP not only induces changes in the RNA secondary structure within a specific region of the EGR1 gene pre-mRNA but also modifies the binding status of the pre-mRNA with U1 snRNP. This suggests that the SNP may alter the RNA conformation, preventing key SFs from binding effectively and thereby affecting the splicing process [76].

### Environmental factors

TME, a complex network of cancer cells, surrounding cells, as well as molecules, is essential to the initiation, development, and spread of tumors. Studying TME-related AS events helps in predicting the prognosis of EC, where numerous SFs and prognosis-related AS events have gradually attracted widespread attention [77].

#### Hypoxia and oxidative stress

Increased oxidative stress frequently coexists with hypoxia in the TME, which concurrently influences tumor resistance to treatment and malignant behavior.

By increasing the expression of SRPKs (CLK1 and SRPK1), changing the intracellular localization of SFs and their capacity to bind to other proteins and pre-mRNA, hypoxia may affect the hyperphosphorylation and expression of SFs (SRSF1, SRSF2, SRSF3, SRSF6, SAM68, HuR, hnRNPA1, hnRNPM, PRPF40B, and RBM4) to adapt to the hypoxic environment and the trend of tumor growth [78–84]. Additionally, it can directly induce AS events. In EC, two distinct non-coding YT521 mRNA isoforms, SNCG isoform2, and anti-angiogenic VEGFA-165b splice variant, are induced by hypoxia to promote tumor progression [85–87]. In glioblastoma, hypoxia stimulates the synthesis of the constitutively active EGFRvIII version of EGFR, which is linked to a poor prognosis [88]. Under hypoxic conditions, the binding of epigenetic factor CCCTC binding factor (CTCF) to intron 1 of BNIP3L promotes autophagy by inducing the production of BNIP3L-F [89].

Oxidants can control a variety of AS events in both cancer cell lines and normal tissues, and change the activity or abundance of SFs. For example, oxidative stress can directly induce multiple exon combinations to be retained in the central variable region of the CD44 gene, the TRA2β4 isoform of exon2 containing multiple PTCs, and SRSF3-TR transcript [90–92].

#### Metabolism

Research has demonstrated a tight relationship between the glycolytic process and Treg cell suppression. Lactate enhances CTLA-4 expression in the TME by promoting the splicing of CTLA-4 RNA via USP39 in a Foxp3-dependent way, which preserves the functional and phenotypic state of tumor-infiltrating Treg cells [93]. However, in human autoimmune disorders, the glycolytic enzyme enolase-1 can cause Foxp3 to produce a version that lacks exon2 (Foxp3-E2), which impairs the suppressive capacity of Treg cells [94].

#### Cytokines

In the variable exon region of CD44 pre-mRNA, transforming growth factor-β (TGF-β) phosphorylates the transcription factor SMAD3 at position T179 to promote the interaction between SMAD3 and the RNA-binding protein PCBP1, inhibiting spliceosome construction and ultimately promoting the synthesis of the mesenchymal-type CD44s [95]. Interleukin-1β (IL-1β) and Interferon-γ (IFN-γ) can influence particular SFs to alter splicing patterns (mostly ES), such as inducing SRSF2 to remove the exon5 of the MHC II gene HLA-DMB, which may impact the autoimmune response [96]. Moreover, IFN-γ can alter the immunopeptidome of melanoma cells, resulting in the formation of novel peptide antigens known as cis-spliced peptides, which are produced by splicing non-contiguous areas inside proteins and may present new targets for cancer immunotherapy [97]. Similarly, to decrease the generation of the anti-apoptotic splicing variant Bcl-xL in K562 leukemia cells, interleukin-6 (IL-6) and granulocyte-macrophage colony-stimulating factor recognize distinct intronic sequences of the Bcl-x gene-encoded pre-mRNA [98]. Interestingly, RNA splicing allows one cytokine to influence the production of another. TGF-β can stimulate SF SFPQ to change the splicing pattern of interferon regulatory factor 1 (IRF1), ultimately lowering downstream IFN-γ expression [99].

## Aberrant RNA splicing and immune regulation

### Tumor cells

**Fig. 3:**
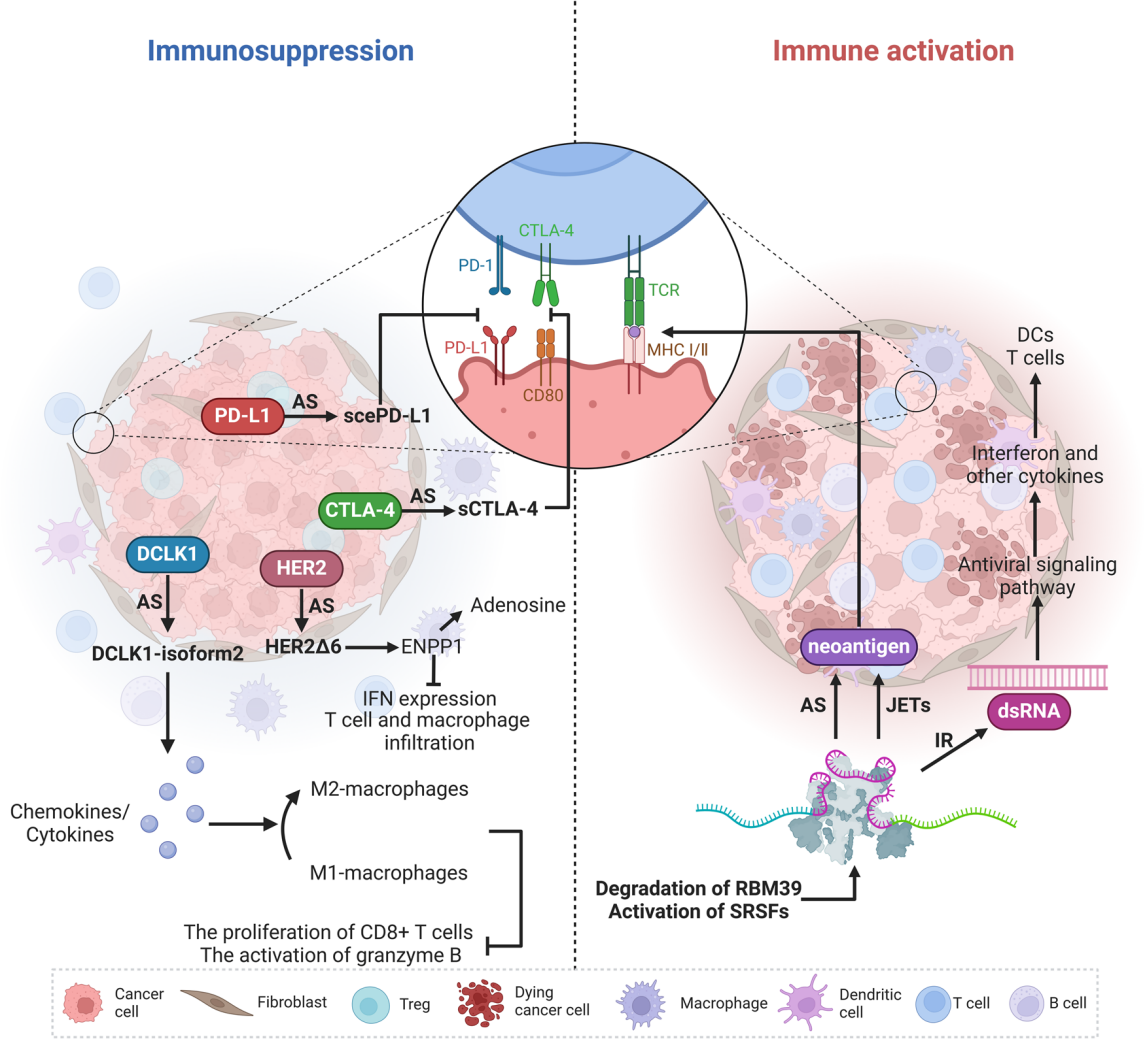
Schematic representation of the positive and negative effects of aberrant RNA splicing on anti-tumor immunity in tumor cells. Created with Biorender.com.

In most cases, aberrant RNA splicing produces more neoantigens in different kinds of cancers than somatic mutations [100, 101]. Interestingly, tumor cells can create tumor-specific peptides via aberrant splicing, which may either promote or inhibit the tumor immune response (Fig. 3 and Table 2).

**Table 2:** Aberrant RNA splicing and immune regulation.

Cell type	Aberrant RNA splicing event	Impact on immune regulation	Reference
Tumor cells	Aberrant splicing of PD-L1 (scePD-L1)	Interferes with PD-1/PD-L1 signaling, aiding immune evasion	[102]
	DCLK1-isoform2	Causes M1 macrophages to shift towards M2, impeding CD8+ T cell growth	[103]
	Aberrant splicing of CTLA-4 (sCTLA-4)	Blocks CD8+ T cell activation	[104]
	HER2Δ6	Lowers IFN expression, produces adenosine with immunosuppressive effects, and inhibits T cell and macrophage infiltration in tumors	[105]
	Activation of SRSFs and degradation of RBM39	Generates novel antigens, enhancing immunotherapy responsiveness	[106, 107]
	JETs (Junctions of Exons to non-coding Transcripts)	Presents new splice variants as antigens, inducing antitumor immune response	[108]
	Intron retention mechanism	Generates dsRNA, triggering antiviral signaling pathways and improving immune surveillance	[109]
Macrophages	MyD88 splice variants (MyD88L and MyD88S)	MyD88S limits immune activation by failing to bind NF-κB	[113]
	Soluble TLR4 (sTLR4)	Suppresses NF-κB signaling and TNF-α generation	[119]
	NLRP3 splice variants (full-length and NLRP3-Δ5)	NLRP3-Δ5 lacks LRR domain, preventing caspase-1 activation	[121]
	FKBP51 splice variant (FKBP51s)	Alters macrophage activity, promoting M2-type activation and inhibiting antigen presentation	[122]
	Acly splice variants (Acly L and Acly S)	Acly S encourages the activation of pro-inflammatory macrophages and the synthesis of inflammatory mediators	[124]
Dendritic cells (DCs)	PKM splice isoforms (PKM1 and PKM2)	PTBP1 controls PKM splicing, affecting T cell activation and recruitment	[126]
	CYLD splice variant (sCYLD)	Induces an overactive phenotype in B cells, Treg cells, and DCs, promoting NF-κB activity	[128–130]
	CXCL16 variant (CXCL16v)	Secreted to recruit immune cells expressing CXCR6	[133]
	DC-CASPIC transcript	Suppresses caspase activity, stimulating NO generation and T cell activation	[134]
B cells	BCR splice isoforms (ΔIgα and ΔIgβ)	Unable to promote IgM translocation, reducing BCR expression	[136, 137]
	BAFF splice variant (ΔBAFF)	Lowers receptor binding capacity, diminishing BAFF’s ability to stimulate B cells	[139]
	Pax-5 splice variants (Pax-5a, b, d, e)	Pax-5b and Pax-5e lack part of the DNA binding domain, affecting transcriptional activation of B cell-specific genes	[140]
	XBP1 splice variant (XBP1-s)	Dual effect on immune regulation	[142–146]
T cells	ST2 splice variant (sST2)	Competitively binds to IL-33, blocking IL-33/ST2L signal transduction	[147, 148]
	CD45 splice isoforms	Differential function in T cell subsets, affecting T cell homeostasis	[153]
	MALT1 splice variants (MALT1A and MALT1B)	MALT1A increases TCR signal transduction, potentially blocked by hnRNPU	[158]
	IRF1 splice variants (full-length IRF1 and IRF1Δ7)	IRF1Δ7 competes with full-length IRF1, lowering *Il12rb1* transcription and IFN-γ expression	[99]
	mCD137 splice variant (sCD137)	Competitively binds to CD137L, blocking CD137-CD137L signal transduction	[161, 162]
	FKBP51 splice variant (FKBP51s)	Affects T cell activation and function, as well as promotes Treg transcription and immunosuppressive ability	[164, 165]
	CD247 splicing variants (CD3ι, CD3θand CD3η)	Cause significant harm to T cell development and alter TCR signaling pathways	[167]

Tumor cells can modify the expression pattern of their own surface antigens through AS to impair the immune system’s ability to recognize and eliminate them. Non-small cell lung tumor cells secrete a splice version of PD-L1 (scePD-L1) that interferes with the PD-1/PD-L1 signaling pathway, assisting tumor cells in avoiding the immune system’s assault [102]. Moreover, tumor-produced splice variants can influence the TME. In PDAC, tumor-produced DCLK1-isoform2 functions as a novel initiator of macrophage polarization, able to cause tumor-associated M1 macrophages to shift towards the M2 type through the secretion of chemokines or cytokines, thereby impeding the growth of CD8+ T cells and the stimulation of granzyme-B, ultimately attaining an immunosuppressive effect [103]. The soluble variant sCTLA-4, which is produced and secreted by the CTLA-4 gene via AS in tumor cells, may block CD8+ T cell activation by interfering with the CTLA-4/CD80 signaling pathway [104]. By upregulating the expression of ectonucleotide pyrophosphatase/phosphodiesterase 1 (ENPP1), HER2Δ6 in breast cancer cells can lower IFN expression, produce adenosine with immunosuppressive effects, and inhibit T cell and macrophage infiltration in tumors [105].

However, through abnormal splicing, tumor cells can also turn “cold” tumors into “hot” tumors, increasing their responsiveness to immunotherapy and triggering immunogenicity. In a solid tumor mouse model, the activation of the SRSF family and the degradation of RBM39 can generate novel antigens mediated by AS, enhancing the effectiveness of immune checkpoint inhibition and stimulating antitumor immunity [106, 107]. The atypical splicing event “JETs” is caused by genomic instability and aberrant expression or mutation of SFs, where irregular splicing connections occur between exons and non-coding areas. When JETs synthesize new splice variants with immunogenic protein sequences, the MHC molecules on the cell surface present them to the immune system as novel antigens, inducing an antitumor immune response [108]. Moreover, abnormal RNA splicing in breast cancer generates double-stranded RNA (dsRNA) in the cytoplasm through an intron retention mechanism. By activating corresponding sensors within the cell, dsRNAs trigger antiviral signaling pathways, encourage the synthesis of interferons and other cytokines, and improve dendritic cell function, T cell activation, and immune surveillance against tumors [109].

### TME

**Fig. 4:**
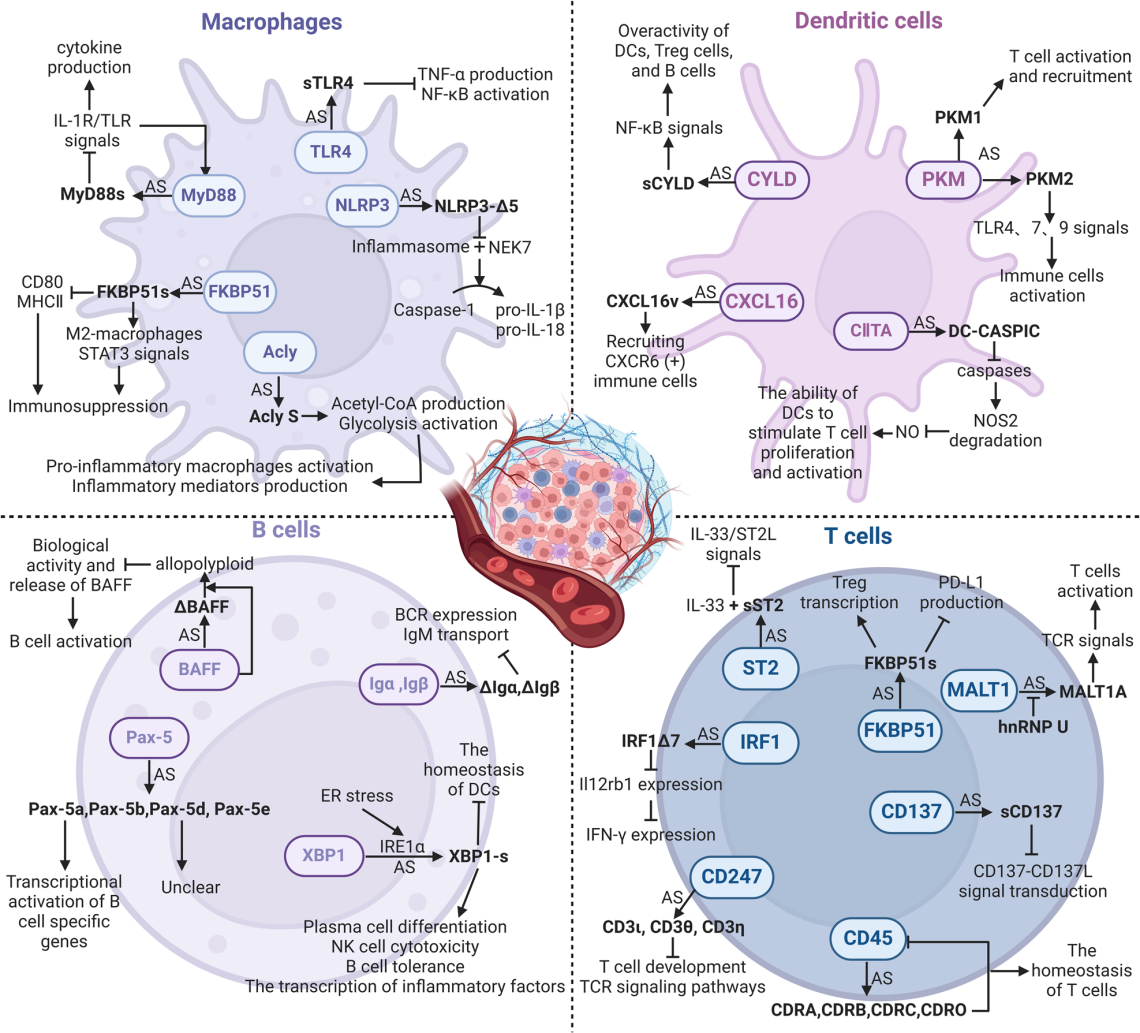
Schematic representation of the impact of AS variants produced by immune cells in the TME on anti-tumor immunity. Created with Biorender.com.

Many cells in TME have been linked to AS events. Lipopolysaccharide (LPS)-stimulated macrophages exhibited 81 AS events [110]. T cell receptor (TCR) stimulation alone in T cells can identify 1319 AS events; co-stimulation of TCR and CD28 in T cells can identify 1575 AS events [111]. Additionally, the development and functional regulation of immune cells largely depend on RNA splicing [110]. Certain splicing isoforms linked to lineage differentiation that are produced by immune cells have been identified by transcriptome analysis [112], but their functions in immune responses, especially in tumor immunity, are not yet clear. Therefore, the following discussion focuses on erroneous RNA splicing and its isoforms that may contribute to tumor immunity in TME cells (Fig. 4 and Table 2).

#### Macrophages

Cytosolic adapter protein MyD88 participates in inflammatory signal transduction downstream of toll-like receptors (TLRs) and IL-1R family members. It has two splice variants: the MyD88L isoform and the MyD88S isoform (lacking exon2). Due to the absence of the intermediate domain necessary for the activation of IL-1R-associated kinases 1 and 4, MyD88S is unable to recruit them, fails to bind to NF-κB, and limits immune activation after the signal [113]. SFs (SF3A, SF3B, Eftud2, SRSF1, and hnRNPU), N6-adenine methyltransferase (METTL3), and TLR signaling pathway components can all control this AS process [114–118].

A key component of the TLR family, TLR4 acts as a pattern recognition receptor (PRR) to identify a range of pathogen-associated molecular patterns (PAMPs), which sets off the TLR4 signaling pathway, causing the release of cytokines and the activation of immune cells. A splice variation of TLR4, soluble TLR4 (sTLR4) has a new exon inserted between exon2 and 3 of its pre-mRNA. This exon has a stop codon, and it has been confirmed to suppress the activation of the NF-κB signaling pathway and the generation of TNF-α driven by LPS [119].

The NLRP3 protein usually interacts with effector caspases and apoptosis-associated speck-like proteins to create the NLRP3 inflammasome complex that can promote the activation of caspase-1, which breaks down precursors IL-1β and IL-18 to enable their maturation and release extracellularly [120]. Human NLRP3 has two isoforms: the full-length variant and NLRP3-Δ5, lacking exon5. Since exon5 encodes the LRR domain involved in the formation of the inflammasome and protein-protein interactions, NLRP3-Δ5 is unable to interact with NIMA-related Kinase 7 (NEK7), ultimately preventing caspase-1 from smoothly activating to process the precursors IL-1β and IL-18 [121].

Immunophilin FKBP51 participates in both cellular stress responses and immunological responses. FKBP51s, a splice variant that alters macrophage activity through many mechanisms, was discovered in the peripheral blood mononuclear cells (PBMCs) of melanoma patients. Firstly, it promotes the STAT3 signaling pathway and macrophage M2-type activation, which are linked to the inhibition of the tumor immune response; secondly, it lowers the expression of MHC II and CD80 to impact macrophage antigen-presenting ability and T cell activation [122].

ATP citrate lyase (ACLY) is an enzyme that synthesizes acetyl-CoA and oxaloacetate (OAA), two important metabolites for cellular metabolism. ACLY promotes tumor immunity by influencing metabolic pathways, including glycolysis and the tricarboxylic acid cycle (TCA cycle) [123]. Acly Long (Acly L) may attach to metabolic substrates in pro-inflammatory macrophages, and protein lactylation modification affects its activity. The immunological regulatory role and metabolic activity of Acly Short (Acly S) differ from those of Acly L because exon14 is absent. The expression of Acly S is linked to increased acetyl-CoA production and glycolysis, which may encourage the activation of pro-inflammatory macrophages and the synthesis of inflammatory mediators [124].

#### Dendritic cells (DCs)

Glycolysis rate-limiting enzyme, pyruvate kinase muscle isozyme (PKM), can generate PKM1 and PKM2 splice isoforms employing mutually exclusive exon9 and 10 [125]. The RNA-binding protein PTBP1 controls the PKM splicing pattern in DCs: a higher level boosts PKM2, while a lower level raises PKM1. DCs lacking PTBP1 promote T cells to activate and recruit in the TME by upregulating the PKM1 variant’s expression [126]. Moreover, elevated PKM2 expression in systemic lupus erythematosus stimulates TLR4, 7, and 9 signaling pathways to activate immune cells [127].

Tumor suppressor CYLD, a ubiquitin-editing enzyme, can negatively regulate the NF-κB and JNK signaling pathways by removing Lys63-linked ubiquitin chains from its target proteins (like TRAF2/6 and NEMO). Due to the deletion of exon7 and 8 that are required for CYLD to bind to TRAF2 and NEMO, sCYLD induces an overactive phenotype in B cells, Treg cells, and DCs, as a positive regulator of NF-κB activity [128–130]. Additionally, it also expands the CD5+ B cell pool abnormally to promote the progression of chronic lymphocytic leukemia (CLL) through the NF-κB signaling pathway [131].

Chemokine CXCL16 participates in immune cell migration and location, and contains a chemokine domain, a transmembrane domain, a mucin-like stalk region, and a cytoplasmic tail [132]. According to its structure, it can bind to cells and act as a soluble chemokine post-separation. Due to the retention of two extra exons and the absence of transmembrane and cytoplasmic domains, the CXCL16v variant present in DCs can be secreted into the extracellular environment to specifically recruit immune cells expressing the chemokine receptor CXCR6 and interact with them [133].

MHC class II transactivator (CIITA) is vital to the antigen presentation process to CD4+ T helper cells. Due to the retention of an extra intronic sequence including a PTC, the DC-CASPIC transcript only has the CARD-like domain. It suppresses caspase activity, limiting the caspase-induced nitric oxide synthase 2 (NOS2) degradation and stimulating NO generation [134]. Furthermore, DCs’ capacity to enhance T cell activation depends on NO, one of the major chemicals generated during DC maturation [135].

#### B cells

Transmembrane proteins Igα and Igβ non-covalently bind to membrane-bound immunoglobulin (Ig) to form the B cell receptor (BCR), facilitating BCR signaling, Ig translocation, and antigen presentation. Through AS, they can synthesize isoforms ΔIgα and ΔIgβ, which are unable to promote the translocation of IgM to the cell membrane due to lacking domains necessary for interaction with Ig [136]. Reduced BCR expression and IgM retention within the endoplasmic reticulum are the results of these isoforms’ heterodimer formation with full-length Igα and Igβ when they are overexpressed [137, 138].

A TNF family member, BAFF, binds to three distinct receptors on B cells to promote them survival, proliferation, and antibody production. ΔBAFF, lacking a 57-bp exon, is co-expressed with BAFF to create a heteropolymer with a lower receptor binding capacity than the homotrimeric complex of BAFF. This can diminish the biological activity and release of BAFF, which in turn can inhibit its ability to stimulate B cells [139].

Transcription factor Pax-5 activates genes involved in B-cell commitment and inhibits genes related to non-B cell lineages in normal B cells. Pax-5a, b, d, and e are the four splice variants identified in mouse spleens and B lymphocyte lines [140]. Since exon2 of Pax-5b and Pax-5e is spliced out, they only contain part of the DNA binding domain and could not directly participate in the transcriptional activation of B cell-specific genes. The novel region that replaces the transcriptional activation domain at the 3’ end of Pax-5d and Pax-5e is yet unknown in its function.

Transcription factor XBP1 is essential to the way cells react to endoplasmic reticulum (ER) stress. IRE1α, a kinase on the ER membrane, changes the splicing patterns to produce the active form XBP1-s when encountering ER stress [141]. Interestingly, XBP1-s has a dual effect on immune regulation. By promoting plasma cell differentiation, augmenting NK cell cytotoxicity, maintaining B cell tolerance, and specifically attaching to the promoters of inflammatory proteins to enhance their transcription, XBP1-s can improve immune responses [142–145]. However, by disrupting dendritic cell homeostasis, XBP1-s can also assist tumor cells in evading immune surveillance [146].

#### T cells

The membrane-bound receptor ST2, often called IL-33R, has a shortened soluble variant, sST2. As sST2 lacks exon9–11 that encode the cytoplasmic and transmembrane domains, it can competitively bind to IL-33 to prevent IL-33/ST2L signal transduction. By blocking IL-33’s pro-inflammatory properties, sST2 slows the growth and metastasis of tumors [147]. Recent research has found that hypoxic colorectal cancer cells can downregulate sST2 through the HIF-IL-33-GATA3 axis, alter the TME, and promote the malignant progression of colorectal cancer [148].

Transmembrane protein CD45 regulates the activity of Src family kinases to control the phosphorylation status of immunoreceptor tyrosine-based activation motifs (ITAMs), thereby initiating the transduction of TCR and BCR signaling pathways [149–151]. By splicing exon4–6 selectively, CD45RABC, RA, RB, RC, and RO isoforms are produced differently in functionally diverse T cell subsets [152]. CD45 is more likely to form homodimers when it skips the variable exons, and because of space restrictions, its intracellular phosphatase activity is suppressed when in the dimeric state. Variable exon4–6 can be made to skip upon T cell activation, which lowers CD45 phosphatase activity and aids in preserving T cell homeostasis [153]. HNRNPLL, HNRNPL, SRSF3, PSF, U2AF26, and Gfi1 can participate in regulating CD45 splicing [154–157].

MALT1 engaged in the transmission of TCR signals to regulate T cell activation and effector function. It has two splice variants: MALT1A, which includes exon7, and MALT1B, which does not. MALT1A is mainly produced by TCR stimulation and can increase TCR signal transduction by enhancing the binding of TNF receptor-associated factor 6 (TRAF6), which can be blocked by hnRNPU, an inhibitor of exon7 inclusion [158].

In malignancies, the previously stated IRF1 can undergo AS to create full-length IRF1 and IRF1Δ7. In order to bind to the *Il12rb1* promoter, IRF1Δ7 competes with full-length IRF1, which would lower *Il12rb1* transcription and subsequently IFN-γ expression. TGF-β has the ability to elevate IRF1Δ7 levels in the TME, interfere with important Th1 cell signaling pathways, and impair its anti-tumor function [99].

CD137 is mostly expressed on the surface of active immune cells, and as a potential target for cancer immunotherapy, it regulates immunological responses, including T cell activation, proliferation, and survival. The binding of CD137 and its ligand CD137L can provide co-stimulatory signals to enhance T cell activation and proliferation, especially for CD8+ T cells [159, 160]. Soluble CD137 (sCD137) is a splicing variant of membrane-bound CD137 (mCD137) that can competitively bind to CD137L to block the CD137-CD137L co-stimulatory signal, potentially leading to immune evasion [161]. In addition, a study has revealed that an increased expression of sCD137 in the blood of lung cancer patients, and those with lower blood levels of sCD137, are more susceptible to achieving a pathological complete response to neoadjuvant immunotherapy [162].

The FKBP51 splicing isoform FKBP51s mentioned above is connected with the immune checkpoint inhibitory signal PD-1/PD-L1, and its coding region and 3’UTR are distinct from FKBP51, leading to shorter protein synthesis [122, 163]. FKBP51s may affect T cell activation and function by affecting PD-L1 folding and maturation, as well as promote Treg transcription and immunosuppressive ability by interacting with the Foxp3 transcription factor, which may influence tumor immune response and immunotherapy efficacy [164, 165].

CD247 (CD3ζ), also known as T cell receptor ζ chain (TCRζ chain), is an essential membrane protein mostly expressed on the surface of T cells and NK cells. When the TCR attaches to an antigen, CD247 interacts with other CD3 chains (CD3γ, CD3δ, and CD3ε), as well as TCRα/β chains, to create TCR-CD3 complexes. These complexes activate T cells through intracellular signaling pathways, resulting in an immunological response to the antigen [166]. Three novel CD247 splicing variants (CD3ι, CD3θ, and CD3η) have been found in mice. The key difference is the C-terminal region, which results in the lack of the third ITAM. Research has demonstrated that these variations have varied impacts on the development and activation of T cells. Specifically, the CD3η variant causes significant harm to T cell development and alters TCR signaling pathways, such as the activation of PLCγ1 and Akt/mTOR signaling pathways [167]. Although no direct studies have been conducted to investigate the impact of CD247 splicing variations on tumor immunity, we may assume that they may indirectly affect tumor immune responses because of their significant influence on T cell development and activation.

## The potential of RNA splicing targets in EC treatment

In addition to being prospective therapeutic targets, cancer-specific splicing variations function as prognostic and diagnostic indicators. Apart from the splicing variants of YT521, SNCG, and VEGF induced by the hypoxic microenvironment previously mentioned, there are other splicing variants in EC that are intimately linked to the incidence and progression of EC (Table 3).

**Table 3:** Splicing variants associated with tumor progression in EC.

Gene	Splice variant	Effect on tumor progression	Splicing regulator	Reference
NF-Y	NF-YAl NF-YAs	In benign tissues. In EC tissues (especially high-grade) and is associated with tumor invasion ability.	Unknown	[175]
FGFR2	FGFR2b FGFR2c	In epithelial cells and is associated with better prognostic factors. In stromal cells and is associated with the aggressive characteristics of tumors.	ESERP1 ESERP2	[176, 177]
CYP24A1	CYP24A1-SV	Promote the proliferation and survival of tumor cells, regulate tumor angiogenesis and influence the infiltration and activity of immune cells.	Calcitriol Progestogens	[178]
p53	Δ40p53	Form aggregates in the cytoplasm and affect the tumor suppressive function of p53	Unknown	[179]
ER	ERαD7 ERβ5	Negatively correlated with tumor grade and stage and positively correlated with disease-specific survival. Increase the sensitivity of ERα(+) EC cells to estrogen.	hnRNPG hTra2-β1 Unknown	[180, 181]
DLX4	BP1	Promote tumor proliferation and migration.	Unknown	[182]
GHRH	GHRH-SV1	Promote cell proliferation and tumor growth.	Unknown	[183]
Survivin	Survivin-ΔEx3 Survivin-2B	Promote the extrinsic apoptotic pathway. Promote the intrinsic apoptotic pathway.	Unknown	[184]
TSNAX and DISCI	TSNAX-DISC1	Promote G1-S phase cell cycle progression and cell proliferation.	CTCF lincRNA-NR_034037	[185]
Numb	Numb-L	Promote tumor growth.	miR-335 RBM10	[186]
TFRC	TFRC-S	Resistance to ferroptosis.	circRAPGEF5 RBFOX2	[172]
RCC1	RCC1-T2 and RCC1-T3	Promote tumor proliferation and migration.	SNORA73B	[187]
FOXM1	FOXM1b and FOXM1c	Promote cell proliferation, migration and invasion, and inhibit apoptosis.	SNORD14E	[173]
ANKHD1	ANKHD1-BP3	Promote tumor metastasis.	HSPB1	[170]

Furthermore, by constructing a network of AS-related SFs and utilizing AS events for genome-wide analysis, many SFs associated with the prognosis of EC have been identified [168, 169]. HSPB1 promotes EC metastasis by downregulating the ANKHD1 splicing variant ANKHD1-BP3 [170]. HnRNPG antagonizes the effect of hTra2-β1 retaining ERα exon7 to reduce ERαD7 that encodes part of the hormone-binding domain, potentially altering the clinical results of the ERαD7 subtype of type I EC [171]. Human EC tissues and cell lines have elevated levels of SF3B1, and when knocked out, it changes many genes’ splicing patterns, including ES and aberrant usage of transcription start and finish sites, etc. Further upstream of the SFs, the overexpression of circRAPGEF5 and SNORD14E in EC affects the SFs RBFOX2 and SRSF1 respectively, and regulates the splicing of exon4 of TFRC and exon7a of FOXM1, thereby affecting the prognosis of EC [172, 173]. Another upregulated SRPK1 in EC has been linked to lower survival rates and regulates RNA splicing by phosphorylating SRSFs, with aberrant activity linked to cancer initiation and progression in diverse tumor cells [29, 174]. Changes in the splicing patterns of several genes linked to cell growth and survival were identified after treating USC cells with the SRPK1 inhibitor SPHINX31, which may have important ramifications for cancer treatment [31]. In order to cure EC, these findings offer possible therapeutic approaches for the creation of inhibitors that target SFs and their upstream molecules.

## Summary and prospects

Exploring the potential targets of RNA splicing contributes to the development of new approaches to cancer diagnosis and therapy. Cancer cells may use aberrant RNA splicing as an intracellular regulation mechanism. It also functions in immune cells, both inside the TME and in normal physiological settings. An in-depth research of splicing events, their regulatory mechanisms, and the different protein variants generated can improve our comprehension of this phenomenon in both healthy and diseased conditions. This will direct the creation of tactics that specifically target the existence and growth of cancer cells or strengthen the anti-tumor immune response, as well as aid in the identification of new therapeutic targets.

EC with MSI-H/dMMR type is the main beneficiary group for immunotherapy. Thankfully, research on how RNA splicing affects the immune response is starting to get traction. Although research on RNA splicing in immune cells is still in its infancy, a deep understanding of the AS events that immune cells undergo in the TME is crucial for optimizing the application of immunotherapy in cancer treatment. In particular, in this field, targeting the new antigenic epitopes produced by RNA splicing or their upstream regulatory molecules for immunotherapy or vaccine development may bring significant benefits to EC patients receiving these treatments.

## Supplementary information

Reproducibility checklist
